# Cost-effectiveness calculators for health, well-being and safety promotion: a systematic review

**DOI:** 10.1093/eurpub/ckab068

**Published:** 2021-05-10

**Authors:** Marja Hult, Olli Halminen, Miika Linna, Sakari Suominen, Mari Kangasniemi

**Affiliations:** 1 Department of Nursing Science, Faculty of Medicine, University of Turku, Turku, Finland; 2 Department of Industrial Engineering and Management, Institute of Healthcare Engineering, Management and Architecture (HEMA), Aalto University, Espoo, Finland; 3 Department of Public Health, University of Turku, Turku, Finland; 4 Department of Public Health, Turku University Hospital, Turku, Finland; 5 Department of Public Health, School of Health Sciences, University of Skövde, Skövde, Sweden

## Abstract

**Background:**

The health, well-being and safety of the general population are important goals for society, but forecasting outcomes and weighing up the costs and benefits of effective promotional programmes is challenging. This study aimed to identify and describe the cost-effectiveness calculators that analyze interventions that promote health, well-being and safety.

**Methods:**

Our systematic review used the CINAHL, PsycINFO, SocINDEX, EconLit, PubMed and Scopus databases to identify peer-reviewed studies published in English between January 2010 and April 2020. The data were analyzed with narrative synthesis.

**Results:**

The searches identified 6880 papers and nine met our eligibility and quality criteria. All nine calculators focussed on interventions that promoted health and well-being, but no safety promotion tools were identified. Five calculators were targeted at group-level initiatives, two at regional levels and two at national levels. The calculators combined different data sources, in addition to data inputted by users. This included empirical research and previous literature. The calculators created baseline estimates and assessed the cost-effectiveness of the interventions before or after they were implemented. The calculators were heterogeneous in terms of outcomes, the interventions they evaluated and the data and methods used.

**Conclusion:**

This review identified nine calculators that assessed the cost-effectiveness of health and well-being interventions and supported decision-making and resource allocations at local, regional and national levels, but none focussed on safety. Producing calculators that work accurately in different contexts might be challenging. Further research should identify how to assess sustainable evaluation of health, well-being and safety strategies.

## Introduction

Interventions that promote health, well-being and safety can contribute to the stability and equality of society and these require people to work across sectors and disciplines.^1^ The impact of these interventions is often difficult to evaluate, due to the vagueness of the concepts and factors that are involved. In addition, the relationships between individual and environmental factors are complex and reciprocal and may result in broad outcomes for society or intangible outcomes.^2^^,^^3^ The influence of such interventions have been questioned, because it can be difficult for external interventions to have an impact on social determinants that strongly influence health status and well-being.^4^

During the last few decades there has been increased interest in evaluating the outcomes of health, well-being and safety promotion interventions. They have been evaluated in relation to morbidity, mortality^5^ and everyday safety.^6^ In addition, evaluating the financial cost of such interventions has made it possible to compare them and apply broad impact measures.^3^^,^^7^ Carrying out a cost-benefit analysis makes it possible to calculate the incremental costs and benefits of an intervention.^8^ The most commonly used economic evaluation methods are cost-utility analyses and cost-effectiveness analyses.^6^^,^^9^ A cost-utility analysis makes it possible to evaluate the wide-ranging health benefits offered by health and well-being promotion interventions, using quality-adjusted life years (QALY). These economic evaluations can then be used to compare different interventions.^10^ Cost-effectiveness analysis is often used to measure specific outcomes in patients with particular diseases, such as life expectancy or medical outcomes. The results depend on the intervention that is chosen, the data that are used, how the costs and outcomes are valued and any discounts that are received.^8^

Even though achieving sustainable outcomes in promoting health, well-being and safety is challenging, it is important to evaluate these in order to make decisions about policies.^11^ Efficient and reliable policy strategies require comprehensive tools that can assess the impact and cost-effectiveness of planned measures.^7^^,^^12^ Digitalization has enabled policy makers, and other bodies involved in evaluating the costs of health, well-being and safety interventions, to develop different calculators that can measure the impact of different policy interventions. In this study, we use the term calculator to refer to tools or frameworks that provide a deterministically constructed outcome and cost estimate for specific interventions that promote health, well-being or safety. These are based on the parameters inputted, or controlled, by the person using the calculator.

A systematic overview of the economic evaluation tools that are available was lacking. The aim of this review was to identify and describe the cost-effectiveness calculators for interventions that promote health, well-being and safety. Our ultimate aim was to provide knowledge for decision-making at local, regional and national levels. We had four research questions: (i) what were the outcomes that the calculators estimated, (ii) what levels of promotion did the calculators target, (iii) what data did the calculators use to evaluate cost-effectiveness and (iv) what methods did the calculators use to estimate the parameters?

## Methods

We conducted a systematic review that followed the Preferred Reporting Items for Systematic Reviews and Meta-Analyses statement,^13^ to identify studies that described cost-effectiveness calculators for interventions that promote health, well-being and safety.

### Data sources and search strategy

**Figure 1:**
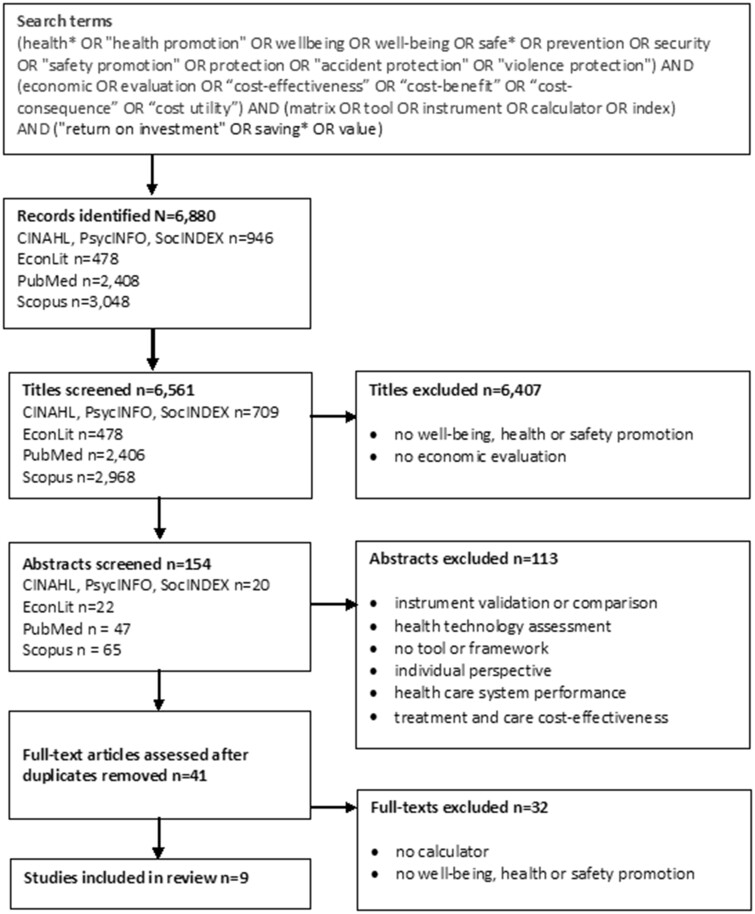
PRISMA flow chart with search terms summarizing how studies were selected

Electronic and manual searches were carried out to identify relevant papers. Electronic searches of previous literature were carried out using the CINAHL, PsycINFO, SocINDEX, EconLit, PubMed and Scopus databases (figure 1). We consulted an information specialist and used the Boolean operators to develop search sentences. The sentences consisted of four groups of terms and their synonyms: (i) health promotion or well-being or safety, (ii) economic evaluation or cost-effectiveness, (iii) instruments or calculators and (iv) return on investment or value. We included studies that were published in English between January 2010 and April 2020 in peer-reviewed scientific journals and had an abstract available. We set the dates based on the preliminary searches. We aimed to identify the most current literature, and therefore years were limited to 2010. The manual search comprised reviewing the reference lists of the papers identified by the electronic search. This search used the same limitations for language and years as the electronic searches.

### Study selection

We selected the studies based on their titles, abstracts and full texts, using pre-defined inclusion and exclusion criteria. Studies were included if they focussed on health, well-being or safety promotion interventions and described calculators that estimated outcomes at local, regional or national levels. These outcomes were cost-effectiveness estimates of the intervention and provided a targeted evaluation of the initiative. We defined health as health promotion and disease prevention,^14^ well-being as social conditions and environments^15^ and safety as preventing injuries in everyday life.^6^ Studies were excluded if they assessed cost-effectiveness at individual levels, validated or compared measurement instruments or focussed on assessing health technology or the performance of service systems. The titles, abstracts and full texts were independently selected by three researchers (M.H., O.H. and M.K.) who then discussed their findings.

The titles of the three first databases were screened by two researchers (M.H. and O.H.) and the titles of the other three databases also by two researchers (M.H. and M.K.). Then the records were circulated so that two researchers (M.H. and M.K.) screened the abstracts from the three first databases and two researchers (M.H. and O.H.) screened the three last databases. We used no reference manager and duplicates were removed after abstract reading in the three researchers’ agreement.

### Data extraction and analysis

First, we read the studies several times to understand the entire data. Then we extracted the data and tabulated the studies according to the author(s), the year of publication, the country, the aim, the tools that were used and the aims and development processes of the calculators (Supplementary table S1) Second, we described the health, well-being and safety promotion interventions (table 1) and grouped the estimated outcomes. Then we identified the methods applied to evaluate the general cost-effectiveness (table 2) and grouped the data in relation to the parameters that were estimated (table 3).

**Table 1:** Targeted promotion interventions assessed by calculators

Activity		Target	
Group level	Individual level disease prevention Improving health and quality of life Reducing risk of illness	Change health behaviour	Smoking^17^^,^^21^^,^^22^^,^^25^
			Alcohol^17^^,^^25^
			Physical activity^17^^,^^21^^,^^25^
			Weight and nutrition^17^^,^^21^^,^^22^^,^^24^^,^^25^
			Mental health and stress^21^^,^^22^^,^^25^
			Sleep^22^
		Screening	Blood glucose^18^^,^^24^^,^^25^
			Blood pressure^18^^,^^25^
			Cholesterol and triglycerides^22^^,^^25^
		Prevent disease	Migraines^22^
			Heart disease^22^
			Diabetes^18^^,^^22^^,^^24^
			Arthritis^22^
Regional level	Health management Improving quality of life and health status Reducing need for hospitalization	Control medication	Medication review to improve glycaemic and glucose control in primary care^18^
			Adherence to prescribed drugs and medical plans for older patients^23^
		Assess and manage care	Triaging patients for follow-up primary care visits^18^
			Replicating, and providing education on, integrated care for chronic diseases, including remote monitoring at regional levels^23^
			Assessing patients with uncontrolled diabetes^18^
			Prevention and early diagnosis of frailty and functional decline in older people^23^
	Advocating well-being and health for ageing people Development and innovation Improving quality of life and health status	Environmental planning	Promoting innovation with regard to age-friendly buildings, cities and environments^23^
National level	Population-level health promotion Reducing mortality and morbidity	Change health behaviour	Breastfeeding^17^^,^^20^
			Cycling^19^

**Table 2:** General description of the cost-effectiveness evaluation methods and data

Study characterization	Calculator development process	Data required by the calculator user
Detailed data-driven calculators for a specific context	Context-specific data-driven empirical estimates were created using individual-level clinical and cost data	The user needed to provide detailed organization-level information to achieve a more detailed analysis of the results^22^^,^^25^
Cost-effectiveness evaluation calculators	Aggregated effectiveness estimates were created based on the literature and statistics	The user needed to provide general-level aggregated information to achieve an estimation of cost-effectiveness of the programme^18–21^^,^^24^
Unified evaluation framework for post-intervention evaluation	A unified framework for post-intervention evaluation of programme cost-effectiveness was created based on standard measures from the literature and industry expert	The user needed to provide the required information on post-intervention health and costs to achieve a cost-effectiveness estimate that was easily compared with similar studies^17^^,^^23^

**Table 3:** Parameter estimation methods used to create the models

Method of parameter estimation	Data employed in model creation	Data application
Empirical research	Individual health status	Risk group detection^22^^,^^24^^,^^25^
	Individual cost of care	Estimate of programme cost-effectiveness^22^^,^^24^^,^^25^
	Organizational-level factors	Contextual control variables^22^
Literature review	Changes in health status	Estimate of programme effectiveness^18–21^^,^^24^
	Cost information	Cost effect, default programme cost information^18–21^
	Organisational characteristics^a^	Default organizational characteristics^18^
User input	Current individual health status	Baseline characteristics^22^^,^^25^
	Current individual health behavior	Baseline characteristics^22^^,^^25^
	Current regional-level health behaviour	Baseline characteristics^19^^,^^20^
	Organizational characteristics^a^	Baseline characteristics^18^^,^^21^^,^^24^
	Personnel cost information	Baseline characteristics^18^^,^^21^
	Health changes after the intervention	Estimate of programme effectiveness^17^^,^^23^
	Cost of the intervention	Estimate of programme effectiveness^17^^,^^23^

a Organizational characteristics typically included the industry type, organization size, number of patients and employees and salary information.

### Quality appraisal

We conducted a quality appraisal for selected studies by modifying the Transparent Reporting of a multivariable prediction model for Individual Prognosis Or Diagnosis statement checklist (TRIPOD), because no such checklist exists for cost-effectiveness calculators.^16^ The TRIPOD checklist consists of 22 items for good reporting of studies. Of this checklist, 10 items that presented every reporting section and were appropriate for calculator studies were selected (Supplementary table S2). The quality appraisal aimed to assess the methodological quality of the original studies, not to exclude studies. The quality appraisal was done independently by two researchers. Based on the quality appraisal, scores ranged from six^17^ to ten^18^ on a scale of 0–10 for individual studies. All the studies reported the background, the aims, the data sources and the interpretation of the results and only two studies described the interventions.

## Results

The electronic searches resulted in 6880 studies (figure 1). The titles were screened and 154 abstracts and 41 full texts were read after duplicates were removed. We selected nine of the studies based on the electronic searches and no more papers were added when we checked their reference lists. Of the nine studies, five were conducted in the USA, two in the Netherlands, one in Australia and one in the UK. All nine studies focussed on health and well-being promotion programmes, but no calculators for safety promotion were identified (Supplementary table S1). Two studies described calculators at the population level,^19^^,^^20^ two at the local level^17^^,^^18^ and two at the group level.^21^^,^^22^

### Outcomes estimated by calculators

Seven of the nine calculators focussed on health and economic outcomes. Two used QALY to quantify the net health benefits,^17^^,^^23^ two used return on investment to evaluate the efficiency of an investment,^17^^,^^24^ and one identified the costs saved by reducing morbidity.^20^ Two calculators estimated health outcomes as incremental health gains in morbidity and reduced mortality.^19^^,^^23^ Three calculators^18^^,^^23^^,^^25^ measured optimal healthcare outcomes as a result of reductions in the use of health and medical care, emergency department visits, readmission rates and optimal pharmacist resources. Three calculators were based on workplace health, well-being promotion and risk reductions interventions and used organizational savings as the key outcome measure. These calculators generated savings by preventing loss of productivity due to staff absenteeism, staff turnover, working while sick, short-term disabilities and medical costs.^21^^,^^24^^,^^25^

To conclude, four calculators assessed savings for employers as reduced disability, absenteeism and claims,^21^^,^^22^^,^^24^^,^^25^ five calculators evaluated savings for society as reduced morbidity and mortality^17^^,^^19^^,^^20^^,^^23^ and reduced medical costs.^18^

### Targeted promotion levels by calculators

#### Group-based calculators

Five calculators focussed on group-level interventions,^21^^,^^22^^,^^24^^,^^25^ (table 1) They aimed to estimate the cost-effectiveness of preventing diseases,^18^^,^^22^^,^^24^ reducing health risks and improving people’s health and quality of life based on changes in health behaviour.^17^^,^^21^^,^^22^^,^^24^^,^^25^ The main emphasis at the group level was on preventing diabetes.^18^^,^^22^^,^^24^ The calculators estimated how health risks could be diminished by reducing smoking and alcohol use^17^^,^^21^^,^^22^^,^^25^ and encouraging weight control and optimal nutrition,^17^^,^^21^^,^^22^^,^^24^^,^^25^ increased physical activity,^17^^,^^21^^,^^25^ better sleep and mental health^22^^,^^25^ and decreased stress at work.^21^^,^^22^ They also included screening to detect and control health risks.^18^^,^^22^^,^^24^^,^^25^

#### Regional-level calculators

Two calculators focussed on regional levels, one for health management^18^ and one for advocacy for health and well-being^23^ (table 1). The health management calculator aimed to treat established diseases by using medication control and assessing and managing care, in order to prevent deterioration and reduce the need for hospitalisation.^18^^,^^23^ Medication control included medication reviews by pharmacists,^18^ and making sure that prescriptions were correctly dispensed and adhered to medical plans.^23^ Care was assessed and managed by triaging, monitoring and early diagnosis in primary care.^18^^,^^23^ Advocating for health and well-being included developing innovative methods for planning age-friendly cities and environments.^23^ Health management and advocacy activities particularly focussed on older people and their general aim was to improve their quality of life and health status.

#### Population-level calculators

Two population-level calculators focussed on establishing and maintaining conditions to minimize health hazards^19^^,^^20^ (table 1), by estimating how changes in health behaviour reduced mortality and morbidity. These included the impact of increased breastfeeding on child morbidity.^20^ They also looked at how increasing cycling reduced deaths at the population-level and the impact of investing in bicycle paths, bicycle parking and traffic calming measures.^19^ Individual breastfeeding support was measured by system-level factors, such as maternity care practices, marketing and nutritional programmes.^20^

### Data for evaluating cost-effectiveness

There were two pre-intervention, data-driven calculators^22^^,^^25^ (table 2). These focussed on context-specific empirical estimates and used individual-level clinical and cost data, such as health risk assessments and medical compensation claims. At the same time, the person who used the calculator needed to provide detailed organizational information on issues like short-term disabilities, to predict the cost-efficiency of programmes more precisely.^22^^,^^25^

There were five pre-intervention cost-effectiveness calculators^18–21^^,^^24^ that used group-level data during their development process. These calculators estimated aggregated effectiveness based on earlier epidemiological data and cohort studies, systematic reviews and statistics. To predict the cost-effectiveness of interventions, the user had to provide group-level aggregated baseline information on the targeted programme population, such as wage statistics and behavioural risk factors.^21^^,^^22^

Two calculators were post-intervention, unified evaluation frameworks,^17^^,^^23^ which had been developed based on previous literature and professional panels.^17^ The person who used the calculator had to enter the post-intervention health and cost information to obtain an estimate that compared the cost-effectiveness to similar studies. These calculator were designed to be used when a health or well-being promotion intervention had already taken place and the data that needed to be used to obtain a cost-effectiveness estimate had already been gathered.^17^^,^^23^

### Methods used to estimate parameters

All nine calculators required users to input key data so that they could estimate the effectiveness of the interventions (table 3) These data provided the baseline parameters^18–22^^,^^24^^,^^25^ that were needed to estimate the effectiveness and costs of the intervention.^17^^,^^23^ These baseline parameters were information on current individual health status, individual and regional health behaviour, organizational characteristics and staffing costs. The data that were required from users to estimate the effectiveness of the intervention were information on changes in health status after the intervention and the cost of the intervention.

Three calculators used data from empirical research to calculate risks at the group level and to estimate the cost-effectiveness of the intervention and the control variables in the context of the intervention.^22^^,^^24^^,^^25^ The data that were used to create the models were information on individual health status and the costs of care and organizational-level factors.

Five calculators employed evidence from scientific literature^18–21^^,^^24^ and this information was used to estimate the average effectiveness of a intervention, the cost-effectiveness as the default value for cost parameters and the default organizational characteristics. The literature reviews that were used to create these parameters ranged from reports from industry experts to systematic scientific reviews.

### Relationship between parameter estimation method and data requirements

The usefulness and applicability of the calculators were linked to the data they used for their estimates. Most of the calculators used existing literature to create the baseline estimates for their models. It appears that the studies that performed their own empirical research to estimate parameters created models that were based on their own specific context or organization.^22^^,^^25^ Alternatively, they were part of a national health promotion programme that had already been evaluated.^24^ There were similarities between the level of detail in the data used to create the evaluation calculators and the data that the user was expected to provide. For example if highly detailed individual level data was used to create the estimates, the same quality of data needed to be provided by the person who used the calculator. Calculators that based their average cost-effectiveness estimates on the existing literature did not expect the user to provide a greater level of detail and aggregate-level information was sufficient. The generic post-intervention evaluation frameworks did not include any prior contextual data in their models. People using these calculators were not expected to provide context-specific data and their contribution was mainly limited to abstract estimates of health benefits and costs, such as QALYs and costs per patient treated.

## Discussion

This review produced new synthesized knowledge on the outcomes, targets and methods of cost-effectiveness calculators. All of the nine calculators we reviewed focussed on health and well-being promotion, but we were unable to identify any calculators that could evaluate safety interventions. The calculators evaluated interventions at group, regional and population levels. Most of the interventions that the calculators evaluated responded to public health challenges by aiming to change individual health behaviour. All the calculators required user input data and combined this with either empirical research or reviews of existing knowledge to create baseline estimates. Most of the calculators evaluated cost-effectiveness at the pre-intervention stage and only two could be used for the post-intervention stage.

It is noteworthy that most of the calculators we identified focussed on evaluating individual health behaviour^17^^,^^21^^,^^22^^,^^24^^,^^25^ in specific contexts.^21^^,^^22^^,^^24^^,^^25^ This raises a question about how useful and applicable these calculators would be in other contexts. Another question is that reviewed studies did not consider equity issues. Calculators used health data mainly from public health and workplace health interventions, and outcomes were not available by different socioeconomic groups, for instance. Calculators for social well-being promotion for the vulnerable groups were lacking. Thus, more attention should be paid to the equality of the interventions that will be measured in the future. For example, taxing alcohol has been presented as an effective strategy^26^ that promotes health and well-being and prevents disease. These types of strategies have the potential to promote both health and equality.

Two important questions that need to be considered are how useful calculators are and how well they can be applied in different countries or contexts than the ones they were developed for. Almost half of the calculators we reviewed were developed for specific contexts.^22–25^ Overall, the applicability of the calculators covered by this review was limited to more affluent countries.^27^ Furthermore, the selected studies did not describe the contents of health and well-being promotion interventions comprehensively. Despite this, the outcome of cost savings was relevant, clearly understood and could be compared with other interventions.^7^

### Practical implications for public administrators

These findings have important implications for public health administrators who are planning to use cost-effectiveness calculators to promote interventions that focus on health and well-being. Our analysis showed that, in order to create a calculator, there had to be scientific cost-effectiveness evidence available before the intervention. If that did not exist then the people who used the calculators had to estimate the cost-effectiveness themselves. However, if a body of evidence already existed on the effectiveness of the intervention, it made it relatively straightforward to create an aggregate-level, cost-effectiveness calculator. In contrast, if a calculator was expected to produce high-level detailed outcomes, the user needed to provide high-level detailed background data and possibly individual level data for the model. This means that it is unlikely that one calculator is suitable for all purposes, as they need to be developed according to particular contexts.

Despite these limitations, cost-effectiveness calculators can guide the design and evaluation of interventions that promote health and well-being and help to improve outcomes. An economic evaluation of interventions should include the type of intervention that the calculator focuses on, the data available from the targeted population and the outcome and cost dimensions.^8^ These should be carefully re-designed if cost-effectiveness is not reached. It should be noted that the calculator models studied by this review did not include probabilistic or other sensitivity analyses that could help policymakers interpret the uncertainty associated with outcomes. However, these analyses are recommended.^28^ Nevertheless, our review provides policymakers with the confidence that calculators that support decision-making are both accessible and modifiable and can be developed for local, regional and national needs.

### Strengths and limitations

The limitations of this review were related to the search strategy and the analysis of the results. In order to make sure that our search terms were comprehensive, we worked with an information specialist and tested several combinations of search terms. For instance, searches covered areas such as health behaviour and disease prevention. However, the terms health, well-being and safety are extensive and abstract concepts, and we could have missed some studies. Furthermore, we found that the terminology for calculators was still fragmented and to some extent undeveloped. Focussing on scientific databases may have caused that we missed some relevant calculators. Also, we included studies only in English, which means that we could have missed some relevant studies. We carried out manual searches of the reference lists of the selected papers and these did not result in any new studies being included in the review. The selected studies were heterogenous, which meant that we could only provide a narrative synthesis of the calculators.

## Conclusion

Our systematic review identified nine cost-effectiveness calculators for interventions that promote health and well-being and between them they supported decisions about resource allocations at local, regional and national levels. Although we included safety promotion in the search, no calculators that covered this area were identified. The calculators were heterogenic in terms of outcomes, the interventions they evaluated and the data and methods used, which made direct comparisons difficult. Depending on the context, and the desired accuracy in terms of outcomes, the calculators were able to assess the cost-effectiveness of interventions either before or after they were implemented. They did this by using various data sources to generate the estimates. There is a need for further research to assess the evidence scientifically and, above all, to create cost-effectiveness tools that cover the fields of health, well-being and safety promotion.

## Supplementary data

Supplementary data are available at *EURPUB* online.

## Funding

This study was funded by the Finnish Prime Minister’s Office (grant number VN/14626/2019).

*Conflicts of interest*: None declared.

**Key points**

- This review identified nine cost-effectiveness calculators that focussed on health and well-being promotion at local, regional and national levels, but none that covered safety promotion.
- The calculators were heterogenic, in terms of outcomes, the programmes they evaluated and the data and methods used, but it is not clear how useful they would be in other contexts.
- The level of accuracy about outcomes depended on the level of background data provided by the calculator and the person using the calculator.

## Supplementary Material

ckab068_Supplementary_Data
