# Supplementary material for: Cost-effectiveness calculators for health, well-being and safety promotion: a systematic review
Source: Eur J Public Health. 2021 May 10;31(5):997–1003. doi: 10.1093/eurpub/ckab068 (PMC8546878; doi:10.1093/eurpub/ckab068)
Supplement: ckab068_Supplementary_Data [file ckab068_supplementary_data.zip › ejph-2020-12-om-1581-File006.docx]

| Supplementary Table 1. Description of the studies that were reviewed and how the calculators were developed. | | |
| --- | --- | --- |
| **Authors, year, country**  **(Quality appraisal)** | **Aim of the study and calculator** | **Calculator development methods** |
| Baxter et al., 2015, Australia  (8/10) | ***Study aims***  To describe the development of a workplace health investment calculator.  ***Calculator***  Workplace Health Savings Calculator  <https://healthierworkplacewa.com.au/learn/>  workplace-health-savings-calculator/  ***Calculator aims***  To develop an evidence-based, simple and easy-to-use workplace health investment calculator that shows annual savings an Australian employer may gain by health and well-being promotion. The tool tends to support employers to engage in workplace health and well-being efforts. | ***Produced by***  A Healthy Workers Initiative project within Population Health Services in the Tasmanian Department of Health and Human Services  ***Development*** ***phases***   1. A literature review identified appropriate effectiveness measures 2. A second review on case studies by PricewaterhouseCoopers established the magnitude of a possible change in estimates 3. Previously developed workplace health calculators were searched and a model produced by the UK’s National Institute for Health and Care Excellence was adapted   ***Data sources***   - PricewaterhouseCoopers’ review of 55 business cases - Australian wage statistics   ***Assumptions***   - Absenteeism would be an employee’s unplanned leave from work - Health promotion programme would be successful when targeted employee’s needs, when participation rates were more than 25%, and senior management and leaders actively supported the programme - Different types of programmes would contribute equally and improved the effectiveness estimates - Programmes produced long-term benefits   ***Performance estimates***   - Worker absenteeism and staff turnover |
| Boehler et al., 2015, the Netherlands  (7/10) | ***Study aims***  To describe the main characteristics and benefits of the Monitoring and Assessment Framework European Innovation Partnership (MAFEIP) tool for estimating the health and economic outcomes of the activities carried out by the European Innovation Partnership on Active and Healthy Ageing.  ***Calculator***  MAFEIP tool  <https://www.mafeip.eu/the-tool>  ***Calculator aims***  To provide an assessment of the likelihood of the anticipated impact that designed interventions will achieve. | ***Produced by***  The European Commissions’ Joint Research Centre, Institute for Prospective Technological Studies, the Directorate General for Communications Networks, Content and Technology and the Directorate General for Health and Food Safety (reference 34 in paper)  ***Development*** ***phases***   1. The monitoring framework representing Triple Win: Quality of life, Sustainability of health and care systems and Innovation and growth 2. Refining and operationalising the initial framework 3. Short-list of candidate indicators for quantifying European Innovation Partnership on Active and Healthy Ageing outcomes 4. Conceptualising a quantitative model 5. Proposing a Markov-Model with three generic health states ('baseline health', 'deteriorated health' and 'death')   ***Data sources***   - The Healthy Life Year statistic (HLY) by Eurostat - A systematic review of studies that provide a link between the HLY and the quality-adjusted life year metric   Epidemiological, economic and effectiveness data from interventions within the European Innovation Partnership on Active and Healthy Ageing  ***Performance estimates***   - Health and economic outcomes as quality-adjusted life years - Health and social care use - Incremental health gain |
| Fishman et al., 2015, the Netherlands  (7/10) | ***Study aims***  To examine the health benefits and health-related economic benefits of cycling in the Netherlands.  ***Calculator***  Health Economic Assessment Tool  https://www.heatwalkingcycling.org/#homepage  ***Calculator aims***  To estimate the value of reduced mortality from a specified amount of cycling or walking. | ***Produced by***  World Health Organization (reference 30 in paper)  ***Development*** ***phases***   1. The project core group commissioned systematic reviews 2. The core group considered the results of these reviews and used them to propose options for, and guidance towards, harmonised methods 3. Practical tool for cycling and walking was developed   ***Data sources***   - A literature review of economic analyses of cycling and walking projects - A review of the value of a statistical life studies - Epidemiological data from two combined Copenhagen cohort studies - A systematic review and meta-analysis on the reduced relative risk of all-cause mortality from regular cycling or walking - Air pollution literature - Literature review on road crashes - Literature on effects on carbon emissions   ***Assumptions***   - Mortality risk would be 10% with 100 minutes of cycling per week - Risk reduction would be controlled for other forms of physical activity and health behaviours - Only people aged 20-65 should be included - A standard value of a statistical life would be $3.6 million for the EU’s 27 countries - A certain proportion of the population would change its mode of transport from an (unknown) average (non-active) transport behaviour to walking or cycling - This average transport behaviour would correspond with the urban background air pollution levels   ***Performance estimates***   - Number and value of deaths per year prevented by cycling |
| Kelly et al., 2010, United States  (9/10) | ***Study aims***  To develop a calculator that measures the potential cost impact of changes in the health risks of Novartis employees and presents the results graphically.  ***Calculator***  Novartis Health Index  ***Calculator aims***  The calculator aims to support health risk management efforts in the workplace, should be based on actual company data, produce a quantifiable output and be useful for communication to senior management. The calculator would link employees’ health risks to company costs and estimate potential cost savings from risk reduction programmes. | ***Produced by***  Novartis company  ***Development*** ***phases***   1. Relationships between health risks and costs were set up (reference 35 in paper) 2. Tool was then developed so that the user could explore alternative scenarios 3. Predictions were scored by comparing the best-case and worst-case scenarios 4. A graphical display of the index was generated   ***Data sources***   - Novartis eligibility - Health risk assessments - Medical claims - Short-term disability - Data on absenteeism and working while sick   ***Assumptions***  High risk in following health issues was defined by the Mayo Clinic health risk assessments:   - Alcohol, emotional health, nutrition, physical inactivity, safety, blood pressure, cholesterol, triglycerides, weight   ***Performance estimates***   - The Novartis Health Index score - Index is a measure of the overall costs associated with the risk profile - Costs were based on medical care, short-term disability, absenteeism and working while sick |
| Lanza et al., 2019, United States  (8/10) | ***Study aims***  To describe validation, evidence-bases, transparent and user-friendly tool kit for policy and decision-makers.  ***Calculator***  Diabetes Prevention Impact Tool Kit  https://nccd.cdc.gov/toolkit/diabetesimpact  ***Calculator aims***  To forecast the economic costs and benefits of implementing a lifestyle change intervention of the National Diabetes Prevention Program. Primary audiences are state health departments, health insurers and employers. | ***Produced by***  The National Diabetes Prevention Program by the Centers for Disease Control and Prevention’s Division of Diabetes Translation (reference 36 in paper)  ***Development*** ***phases***   1. Designing a simulation model 2. Developing a detailed technical report for transparency 3. Implementing recommendations from user testing 4. Displaying customised health and economic impact results   ***Data sources***   - National Health and Nutrition Examination Survey (2011-2014) - Behavioral Risk Factor Surveillance Survey (2014)   ***Assumptions***   - Predicted prevalence for three risk groups - Default group were people with prediabetes with specified plasma glucose levels - User could also select lower or higher risk group - The groups only included adults without diabetes or undiagnosed diabetes and a body mass index of 24 kg/m^2^ or higher   ***Performance estimates***  The tool estimated programme costs, diabetes-related medical costs and return on investment, along with other costs and health outcome measures. The tool reported savings from prevented productivity loss that was based on absenteeism. |
| Lister et al., 2013, United Kingdom  (6/10) | ***Study aims***  To report the development of tools that evaluate the value for money of behaviour change investments.  ***Calculator***  Behavior Change Evaluation Tools  ***Calculator aims***  To provide quantitative measures of value for money gained by health behaviour change for local government councils. | ***Produced by***  The UK Department of Health commissioned a programme led by the National Social Marketing Centre  ***Development*** ***phases***   1. Current guidelines and studies reviewed, with input from expert panel, on evaluation of interventions to support behaviour change for better health 2. The fields of behaviour change were identified: smoking cessation, alcohol harm reduction, obesity reduction, breastfeeding continuation and bowel cancer survey responses 3. Tools were developed as Excel applications using 4 to 6 worksheets   ***Data sources***   - Evidence from the National Institute for Health and Clinical Excellence and Health England - Data produced with the National Burden of Disease Tool of the World Health Organization   ***Assumptions***   - Life expectancy for a healthy person was assumed to be 81 years - Changes in health outcomes and cost savings would be compared with ongoing trends, assuming a continuation of current policy measures - After 10 years the health gain achieved by behavioural change would end - The social value of would be £25.000 in 2007/2008 prices   ***Performance estimates***   - Discounted costs and benefits as costs per QALY - Social return on investment - Theoretical annual health and cost savings due to the impact of behavioural changes - Impact of behaviour changes on clients, employers and Government |
| Sacro et al., 2019, United States  (10/10) | ***Study aims***  To describe the development of a population health pharmacist (PHP) value calculator, to demonstrate its value to stakeholders and to discuss the use of the pharmacist value calculator to engage pharmacy, clinical, administrative and financial leaders.  ***Calculator***  PharmValCalc  ***Calculator aims***  To forecast required PHP resources, the impact of pharmacists on preventable, medication-related hospital and emergency visits, improvement in glycaemic control and uncontrolled hypertension and the financial impact of PHP interventions. | ***Development*** ***phases***   1. Defining PharmValCalc’s purpose 2. Selecting population health pharmacist (PHP) interventions 3. Identifying PharmValCalc audience 4. Defining value provided by pharmacists 5. Selecting PHP care delivery model 6. Determining PHPs impact on value 7. Building PharmValCalc 8. Validating PharmValCalc   ***Data sources***   - Literature search on PubMed   ***Assumptions***   - Model 1 (low-intensity PHP model) - Model 2 (high-intensity PHP model)   ***Performance estimates***   - Pharmacist resources - Reduction of medication-related, 30-day hospital readmission rates - Reduction of medication-related, emergency department visits - Increase in the percentage of patients with haemoglobin A1c values < 9% or blood pressure values < 140/90 mmHg |
| Schwatka et al., 2019, United States  (9/10) | ***Study aims***  To describe and evaluate a web-based calculator that communicates the value of investing in employee health and well-being to prevent work-related injuries, illnesses and fatalities.  ***Calculator***  Health Risk Calculator  www.ucdenver.edu/chwe/calculator  ***Calculator aims***  To educate employers on the relationship between health risks and workers’ compensations costs (medical care and indemnity compensation). | ***Produced by***  Pinnacol Assurance  ***Development*** ***phases***   1. Algorithm development 2. Coding the web application 3. Tool evaluation with the Reach Effectiveness Adoption Implementation programme evaluation framework   ***Data sources***   - The Pinnacol Assurance Health Risk Management study (2010-2014) included 25,993 health risk assessments from 16,926 employees - Data on worker compensation claims data from Pinnacol Assurance   ***Assumptions***   - 34 health-related predictor variables that were shown to be associated with health care costs - An average claim cost was obtained from Pinnacol Assurance’s entire portfolio of businesses   ***Performance estimates***  Number and costs of claims |
| Stuebe et al., 2017, United States  (9/10) | ***Study aims***  To determine the impact of changes in breastfeeding rates on population health.  ***Calculator***  Breastfeeding Savings Calculator  <http://www.usbreastfeeding.org/p/cm/ld/fid=439>  ***Calculator aims***  To estimate changes in disease burden following changes in rates of breastfeeding from birth to 12 months and rates of exclusive breastfeeding at six months at the population level. | ***Produced by***  The Institutional Review Board of the Cambridge Health Alliance  ***Development*** ***phases***   1. Monte Carlo simulation with a cohort of women who were aged 15 in 2002 and their children 2. Calculator which estimates the effect of changes in breastfeeding rates across the first year of life was constructed 3. The effect of changes in breastfeeding rates was determined for each of the 50 states in the United States   ***Data sources***   - Cohort of women aged 15 in 2002 and their children - Breastfeeding and disease outcomes from the previous literature - National-level and state-level data for breastfeeding initiation, exclusive breastfeeding at 2 days, 3 months, and 6 months, and any breastfeeding at 6 and 12 months - Census data of 15-year-old women in each state in 2002 - 2012 vital statistics data on overall births and the number of very low birth weight deliveries in each state   ***Assumptions***  The health impact of a change in breastfeeding rates would be additive.  ***Performance estimates***  The impact of a 10% change in breastfeeding rates on cases and costs of five maternal and nine child diseases. |
