# Supplementary material for: Cost-effectiveness calculators for health, well-being and safety promotion: a systematic review
Source: Eur J Public Health. 2021 May 10;31(5):997–1003. doi: 10.1093/eurpub/ckab068 (PMC8546878; doi:10.1093/eurpub/ckab068)
Supplement: ckab068_Supplementary_Data [file ckab068_supplementary_data.zip › ejph-2020-12-om-1581-File007.docx]

| Supplementary Table 2. Quality appraisal using modified TRIPOD checklist.^6^ | | | | | | | | | |
| --- | --- | --- | --- | --- | --- | --- | --- | --- | --- |
|  | Baxter et al. | Boehler et al. | Fishman et al. | Kelly et al. | Lanza et al. | Lister et al. | Sacro et al. | Schwatka et al. | Stuebe et al. |
| 1. Is the context and rationale for developing or validating the tool, including references to existing tools, explained? | Y | Y | Y | Y | Y | Y | Y | Y | Y |
| 2. Are the objectives, including whether the study describes the development or validation of the tool or both, specified? | Y | Y | Y | Y | Y | Y | Y | Y | Y |
| 3. Is the source of data separately for the development and validation data sets, if applicable, described? | Y | Y | Y | Y | Y | Y | Y | Y | Y |
| 4. Are details of intervention received given? | N | N | N | N | N | N | Y | N | Y |
| 5. Is the outcome that is predicted by the tool, including how and when assessed, clearly defined? | Y | Y | Y | Y | Y | N | Y | Y | Y |
| 6. Are all predictors used in developing the tool, including how and when they were measured, clearly defined? | Y | N | Y | Y | N | N | y | Y | Y |
| 7. Is an overall interpretation of the results, considering objectives, limitations, results from similar studies, and other relevant evidence, given? | Y | Y | Y | Y | Y | Y | y | Y | Y |
| 8. Are the limitations of the study discussed? | Y | N | Y | Y | Y | N | Y | Y | Y |
| 9. Is the potential use of the tool and implications for future research discussed? | N | Y | N | Y | Y | Y | Y | Y | N |
| 10. Is the source of funding and the role of the funders for the present study given? | Y | Y | N | Y | Y | Y | Y | Y | Y |
| Total score | 8 | 7 | 7 | 9 | 8 | 6 | 10 | 9 | 9 |
| Y = Yes, N = No |  |  |  |  |  |  |  |  |  |
